# The impact of zero markup drug policy on patients' healthcare utilization and expense: An interrupted time series study

**DOI:** 10.3389/fmed.2022.928690

**Published:** 2022-11-15

**Authors:** Zheng Zhu, Junfeng Wang, Yan Sun, Jiawei Zhang, Peien Han, Li Yang

**Affiliations:** Department of Health Policy and Management, School of Public Health, Peking University, Beijing, China

**Keywords:** hospitalization, expenditures, utilization, interrupted time series analysis, zero markup drug policy

## Abstract

**Objective:**

To curb the unreasonable growth of pharmaceutical expenditures, Beijing implemented the zero markup drug policy (ZMDP) in public hospitals in 2017, which focused on separating drug sales from hospital revenue. The purpose of this study is to evaluate the impacts of ZMDP on healthcare expenditures and utilization for inpatients.

**Methods:**

The Beijing claims data of inpatients diagnosed with ischemic heart disease (IHD), chronic renal failure (CRF), and lung cancer (LC) was extracted from the China Health Insurance Research Association (CHIRA) database. The study employed an interrupted time series to evaluate the impacts of ZMDP on healthcare expenditures and utilization.

**Results:**

The changes in total hospitalization expenses, health insurance expenses, and out-of-pocket expenses were not statistically significant neither in level change nor in trend change for inpatients diagnosed with IHD, CRF, or LC after implementing ZMDP (all *P* > 0.05). The Western medicine expenses for the treatment of inpatients diagnosed with IHD significantly decreased by 1,923.38 CNY after the reform (*P* < 0.05). The Chinese medicine expenses of inpatients diagnosed with CRF instantaneously increased by 1,344.89 CNY (*P* < 0.05). The service expenses of inpatients diagnosed with IHD and LC instantaneously increased by 756.52 CNY (*p* > 0.05) and 2,629.19 CNY (*p* < 0.05), respectively. However, there were no significant changes (*P* > 0.05) in out-of-pocket expenses, medical consumables, imaging, and laboratory test expenses of inpatients diagnosed with IHD, CRF, or LC. The initiation of the intervention immediately increased the number of inpatient admissions with LC by 2.293 per month (*p* < 0.05).

**Conclusions:**

The ZMDP was effective in reducing drug costs, and the effects on healthcare utilization varied across diseases type. However, the increase in medical service and Chinese medicine expenses diminished the effect of containing healthcare expenses and relieving the financial burdens of patients. Policymakers are advised to take multiple and long-lasting measures, such as provider payment methods reform, volume-based drug procurement, and drug price negotiation to improve the affordability of patients thoroughly.

## Introduction

In the era of planned economy, every Chinese citizen could enjoy free medical services, and public healthcare facilities were supported by government subsidies (1). However, the Chinese government was unable to sustain such a heavy financial burden for a long period. Since 1950, a 15% makeup on the price of drugs had been allowed (2, 3). After the economic reform in the early 1980s, the proportion of direct government financing dropped to an average of <10% of public hospital budgets, which created significant self-funding financial pressures within public hospitals. Public hospitals were given inappropriate incentives and encouraged physicians to prescribe superfluous drugs by linking their income with the monetary values of the drugs they prescribed (4). This phenomenon resulted in a rapid increase in health expenditure and inappropriate treatment (5–7). In the two decades before the essential medicine reform, total pharmaceutical expenditures accounted for an average of 45.7% of total health expenditures (8). Extremely high drug expenses led to public discontent over unaffordable healthcare and seriously affected the relationship between doctors and patients (9).

To curb the unreasonable growth of pharmaceutical expenditures, the Chinese government launched zero mark-up drug policy (ZMDP) in 2009, which aimed to discourage healthcare providers from overprescribing drugs and improve the affordability of healthcare. In response to the central government's call for public hospital reform, five tertiary hospitals in Beijing were chosen as pilot hospitals to implement the ZMDP in 2012. On April 8, 2017, Beijing implemented comprehensive healthcare reform in all public hospitals, which focused on separating drug sales from hospital revenue (10). The previously allowed 15% mark-up from drug sales was removed in all public to cut off the economic interests from drug sales. In addition, the reform adjusted the prices of 435 medical services, with increased prices for surgery and traditional Chinese medicine which reflect the professional value and require experience and labor, and decreased prices for computed tomography and magnetic resonance imaging (11).

Previous literature that examined the impact of this policy on healthcare utilization and expenditures has not drawn consistent conclusions about the ZMDP. Some studies found that ZMDP curbed the increase in hospitalization expenses (3, 10) and reduced the economic burden on patients (12). The policy also contributed to optimizing the cost structures to a certain degree without adversely impacting the operation of the hospitals (13). However, some studies suggested that there was no significant change in total expenses after the ZMDP (14, 15), and it was not an effective way to control healthcare expenditure (15–17). Furthermore, the policy may not be enough to eliminate the financial incentives of healthcare providers (15, 17). The significant decrease in drug expenditure resulted in more patients' healthcare needs induced by healthcare providers (17, 18). According to previous studies, it was difficult to obtain a consistent conclusion. In this study, we selected the inpatients diagnosed with IHD, CRF, and LC for analyses. On the one hand, all three diseases imposed heavy burdens on patients and their families, as well as China's economy and the healthcare system. The age-standardized IHD and LC were among the top three leading causes of Years of Life Lost (YLLs) in 2017 (19). CRF similarly had an enormous impact on the health care system in China. Dialysis expenses of 1.59 trillion RMB in 5 years equaled 1.3 times the annual government health expenditure in 2015 (20). On the other hand, the reform in Chongqing (another metropolitan city of China) triggered protests by hundreds of patients with CRF undergoing dialysis within only 6 days of implementation, due to the higher out-of-pocket payments, and consequently resulted in the failure of that reform (21). It is crucial to investigate whether the reform in Beijing resulted in similar problems. Furthermore, the lack of appropriate and detailed data has led most studies to be limited in the analysis of the comprehensive impact of ZMDP. A more comprehensive and detailed assessment was warranted to evaluate the impact of ZMDP. To fill this gap, based on the Beijing claim database from 2016 to 2018, the study investigated the impact of ZMDP on healthcare expenditures and utilization for inpatients. This study used more detailed individual-level data to analyze the changes in various components of expenses and healthcare utilization. It presented new empirical evidence of the ZMDP, and explore the remaining challenges and the lessons learned.

## Materials and methods

### Study setting and intervention

The study was conducted in Beijing, the capital of China, with 21.7 million inhabitants. Beijing ranked first in China in terms of GDP per capita at 128,927 yuan in 2017. It has 116 tertiary hospitals whereby the abundance of medical resources exceeds that of other cities in China (11). In 2016, revenues from drug sales accounted for more than 45.1% of total revenues in tertiary public hospitals in Beijing, while the national average was 38.9% (22). Starting in April 2017, Beijing municipal government implemented a comprehensive public hospital reform that removed previously allowed 15% mark-up from drug sales. Concurrently, the prices of 435 medical services were adjusted. The intervention was integrated into the health system. Therefore, the study assessed the effect of the overall policy and programmatic changes that combine ZMDP with adjustment of medical services prices, rather than the single effect of ZMDP.

### Study design

The interrupted time series (ITS) design was employed to evaluate the changes in healthcare utilization and expenses of patients diagnosed with IHD, CRF, and LC. This study used the timing when the ZMDP was first implemented on April 8, 2017 as the intervention point. The pre-ZMDP period was defined as the period from January 1, 2016 to April 8, 2017, while the post-ZMDP period was defined as the period from April 9, 2017 to December 31, 2017.

### Data sources

The study extracted the Beijing claims data from the China Health Insurance Research Association (CHIRA) database, which is a medical insurance management information system initiated in 2007 that contains nationwide consecutive inpatient and outpatient visit claims data in China (23). The CHIRA data are collected annually from the local insurance centers of a random de-identified sample of selected areas of China including at least 2% of patients for municipalities (24). The database contains patient basic information, diagnosis results, healthcare service utilization, and healthcare expenditure details. In this study, the inpatients diagnosed with IHD, CRF, and LC between January 1, 2016 and December 31, 2017 were chosen for analysis.

### Indicators

Inpatient healthcare expenditure and utilization were the outcomes of interest. Healthcare expenditures were divided into drug, diagnosis, laboratory, medical consumables, and medical services expenses per inpatient admission. Healthcare utilization was measured by the number of inpatient admissions and the usage of consumables, imaging, lab tests, and service.

### Statistical analysis

The interrupted time series (ITS) design is a valuable and commonly used quasi-experimental design to assess the impact of a health policy change in low- and middle-income health systems (25, 26). The study employed segmented regression for model parameterization in the analysis of ITS designs, which includes two linear segments with one trend pre-intervention and one trend post-intervention (25). The study used the three usual ITS components related to the pre-intervention slope, the level of change, and the change in slope in the post-intervention period (26). Usually, the regression model can be formulated as follows:


(1) $$\begin{array}{l} Y_{t}=\beta_{0}+\beta_{1}\;*\;X_{t}+\beta_{2}X_{ZMPD}+\beta_{3}\;*\;X_{t}X_{ZMPD}+e_{t} \end{array}$$

Where Y_t_ is the outcome variable at time t. X_t_ is the continuous-time variable that refers to time from the start of the observation. This study takes 2 weeks as a unit. X_ZMDP_ is a binary dummy variable, where the period before the intervention is represented by 0, and those after are represented by 1. This study used the period when the ZMDP was first implemented on April 8, 2017 as the intervention point. β_0_ represents the baseline level of the outcome variable at the beginning of the observation period. β_1_ estimates the underlying pre-intervention trend (the change in Y associated with a single-unit increase in time before the intervention). β_2_ estimates the instantaneous level change following the ZMDP. β_3_ is purported to represent the differences between pre-and post-intervention slopes. e_t_ is the random error value. The study tested for autocorrelation using Durbin-Watson tests, autocorrelation, and partial-autocorrelation function plots and applied Newey-West standard error model to address autocorrelation and heteroscedasticity.

Stata version 16.0 was used to perform the ITS analysis.

## Results

The incidence rate of IHD, CRF, and LC was 246.09, 217.86, and 58.56 per 100 k, respectively in China in 2019 (27). Table 1 summarized the descriptive results for inpatients. In the present study, 3,478 inpatients across 24 months were enrolled, including 2,680 inpatients diagnosed with IHD, 391 with CRF, and 407 with LC. Most inpatients use urban employee basic medical insurance (UEBMI) for payment, accounting for 94.13% of the total. Inpatients were mainly treated in tertiary hospitals, with little change after the reform.

**Table 1:** Basic characteristics of inpatients.

	**IHD**		**CRF**		**LC**	
**Category**	**Pre-ZMDP**	**Post-ZMDP**	**Pre-ZMDP**	**Post-ZMDP**	**Pre-ZMDP**	**Post-ZMDP**
**Insurance**						
Residents	89	88	7	10	5	5
Employees	1,180	1,323	173	201	234	163
**Gender**						
Male	761	884	97	124	163	95
Female	508	527	83	87	76	73
**Levels of care**						
Tertiary	1,000	1,106	159	191	213	157
Secondary	269	284	21	14	26	11
Primary	0	21	0	6	0	0
Age	66.18	67.23	64.43	61.09	65.28	65.53
Length of stay	9.1	9.6	16.3	13.8	13.4	12.6
Admissions	1,269	1,411	180	211	239	168

### The impacts on hospitalization expenses

As can be seen from Table 2, the changes in total hospitalization expenses, health insurance expenses, and out-of-pocket expenses were not statistically significant neither in level change nor in trend change for inpatients diagnosed with IHD, CRF, or LC (*P* > 0.05) after implementing ZMDP.

**Table 2:** Impacts on hospitalization expenses (ITS model).

**Category**	**Coefficient**	**Total**	**Insurance**	**Out-of-pocket**
IHD	β1	−12.70	−4.85	−15.07
	β2	−1,304.91	−924.14	172.79
	β3	355.73^*^	279.26^*^	58.50
	β0	29,469.02^***^	23,981.65^***^	4,260.24^***^
CRF	β1	−25.48	−68.91	−2.41
	β2	−374.61	−566.42	−268.96
	β3	−159.24	4.60	8.75
	β0	25,056.08^***^	22,886.57^***^	1,061.95^***^
LC	β1	9.09	6.84	8.23
	β2	227.10	770.07	−1076.50
	β3	149.53	46.19	123.85
	β0	34,706.31^***^	28,114.30^***^	3,214.40^***^

Significance codes:

*** *p* < 0.01,

^**^p < 0.05,

* *p* < 0.1.

### The impact on detailed inpatients expenses

**Figure 1:**
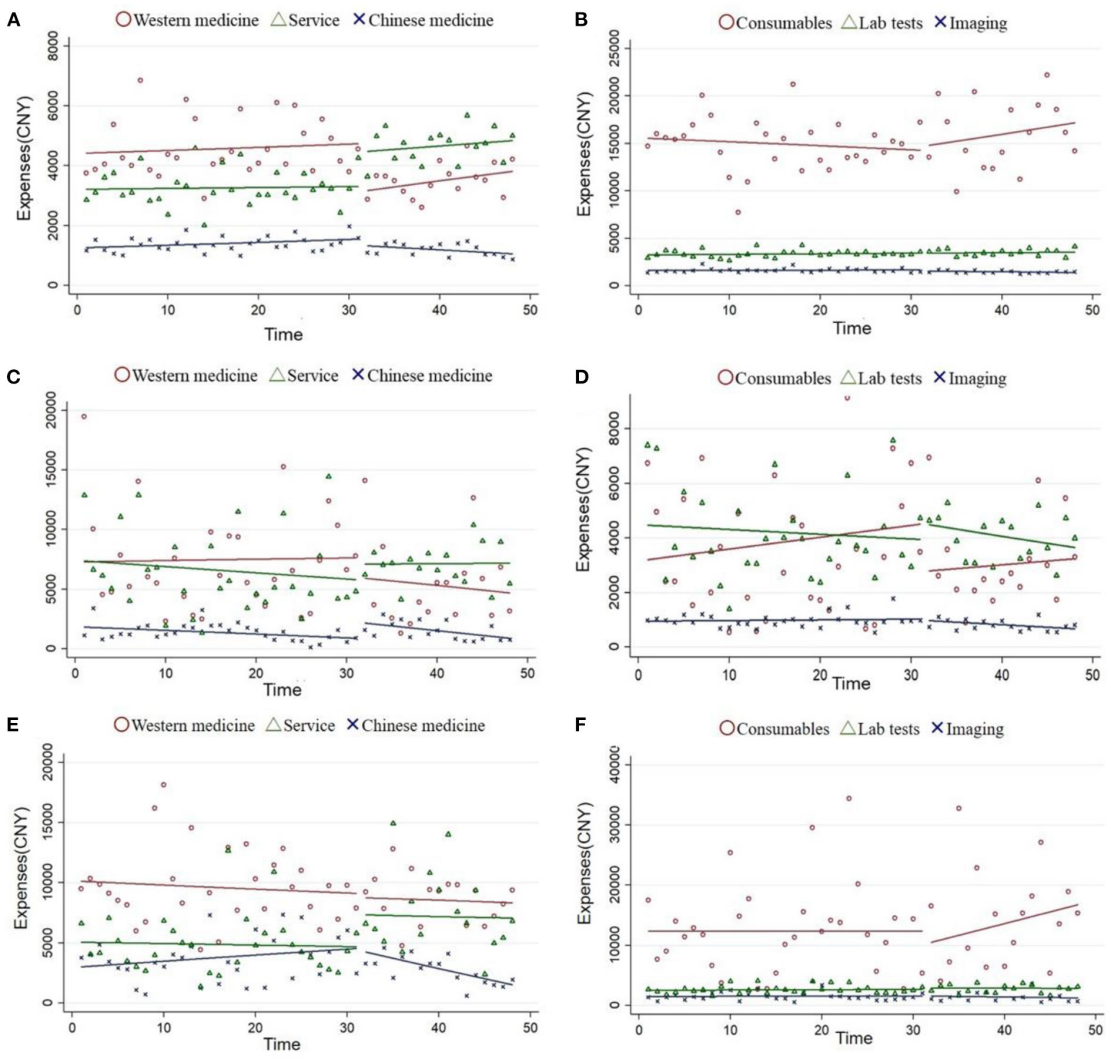
Changes in disaggregated hospitalization expenses (ITS model). Ischemic heart disease **(A,B)**; chronic renal failure **(C,D)**; and lung cancer **(E,F)**.

As shown in Table 3 and Figure 1, the Western medicine expenses of inpatients diagnosed with IHD significantly decreased by 1,923.38 CNY after the reform (*P* < 0.001). The Chinese medicine expenses of inpatients diagnosed with CRF instantaneously increased by 1,344.89 CNY (*P* < 0.05). However, there was a significant long-term trend of decreasing Chinese medicine expenses in patients with IHD and LC after ZMDP (*P* < 0.05).

**Table 3:** Impacts on detailed hospitalization expenses (ITS model).

**Category**	**Coefficient**	**Western medicine**	**Chinese medicine**	**Consumables**	**Imaging**	**Lab tests**	**Service**
IHD	β1	10.44	9.46	−41.90	0.66	5.38	3.01
	β2	−1,923.38^***^	−206.82	221.49	−132.39	−51.48	756.52^*^
	β3	84.45^*^	−25.98^**^	211.13	−6.92	12.00	84.09^**^
	β0	4,405.60^***^	1,255.18^***^	15,596.32^***^	1,656.44^***^	3,283.05^***^	3,218.04^***^
CRF	β1	12.30	−31.13^**^	43.26	2.90	−17.54	−51.95
	β2	−1,644.57	1,344.89^***^	−1,732.98	−45.29	606.52	1,323.58
	β3	−88.46	−49.02	−15.17	−21.27	−35.63	54.85
	β0	7,274.99^***^	1,880.86^***^	3,160.99^***^	943.45^***^	4,485.33^***^	7,416.19^***^
LC	β1	−34.20	50.97	−1.96	1.15	4.67	−11.54
	β2	−340.07	−161.72	−2,203.51	53.98	249.08	2,629.19^**^
	β3	10.13	−219.66^***^	391.95	−18.72	−9.43	−4.71
	β0	10,167.94^***^	2,986.83^***^	12,400.93^***^	1,534.69^***^	2,545.40^***^	5,070.50^***^

Significance codes:

*** *p* < 0.01,

** *p* < 0.05,

* *p* < 0.1.

It was observed that the service expenses of inpatients diagnosed with IHD and LC instantaneously increased by 756.52 CNY (*p* < 0.1) and 2,629.19 CNY (*p* < 0.05), respectively. The slope of the service expenses of inpatients diagnosed with ischemic heart significantly declined in the post-ZMDP period (*P* < 0.05). However, there were no significant changes (*P* > 0.05) in medical consumables expenses, imaging expenses, and laboratory test expenses of inpatients diagnosed with IHD, CRF, or LC.

### The impact on healthcare utilization

As can be seen from Table 4, the initiation of the intervention immediately increased the number of inpatient admissions with LC (β_2_ = 2.293, *p* < 0.05). The number of inpatient admissions with IHD or CRF was not statistically significant neither in level change nor in trend change (all *P* > 0.05).

**Table 4:** The impact on the number of inpatient admissions (ITS model).

**Coefficient**	**IHD**	**CRF**	**LC**
β1	0.218	0.052	0.029
β2	−4.585	−1.859	2.293^***^
β3	0.736	0.183	−0.121^***^
β0	70.077^***^	10.071^***^	8.073^***^

Significance codes:

*** *p* < 0.01,

^**^p < 0.05,

^*^p < 0.1.

Analysis of the utilization of inpatient services is shown in Table 5. The medical consumables usage of inpatients diagnosed with IHD and LC significantly decreased (*p* < 0.05), but the change in long-term trends was not statistically significant (*p* > 0.05). There was no significant change in the utilization of laboratory tests and service items for inpatients diagnosed with IHD, CRF, or LC (*p* > 0.05).

**Table 5:** The impact on the number of inpatients services (ITS model).

**Category**	**Coefficient**	**Consumables**	**Imaging**	**Lab tests**	**Service**
IHD	β1	0.21	0.01	0.15	0.23
	β2	−114.00^***^	0.54	−6.78	−13.65
	β3	2.52	−0.11	0.78	4.06
	β0	185.19^***^	15.32^***^	126.29^***^	280.38^***^
CRF	β1	−5.28^**^	0.01	−0.34	−6.57^*^
	β2	−100.59	2.79	13.14	37.48
	β3	5.01	−0.30^*^	−2.22	3.93
	β0	371.27^***^	11.77^***^	183.60^***^	539.42^***^
LC	β1	−1.202	0.059	0.118	−4.009^*^
	β2	−81.985^**^	−0.183	16.668	60.807
	β3	−1.125	−0.232	−1.250	−5.351
	β0	241.768	9.688	94.265	446.932

Significance codes:

*** *p* < 0.01,

** *p* < 0.05,

* *p* < 0.1.

## Discussion

The rapid rise in medical costs is a common problem faced by most countries, and it is also one of the challenges to be addressed in China's healthcare system reform (4, 28). In order to control the rapid growth of medical expenditures and address the distorted cost structure, Beijing implemented the comprehensive reform of separating medicine from medicine on April 8, 2017, eliminating the 15% markup on drug sales and adjusting the prices of 435 medical services (13).

Using more detailed individual-level data, the present study found that the ZMDP policy was not associated with the decrease in total hospitalization expenses and Out-of-pocket expenses, which is consistent with a previous study conducted in Beijing (13) and also with studies in other parts of China (15–17). In particular, Chen et al. suggest that total expenses increased by 6.88% per patient after the implementation of ZMDP in Shanghai, while out-of-pocket expenses increased by 11.27% (16). The challenges of inflated medical expenses have not been adequately dealt with (29).

In order to further explore the result, this study used more detailed data to evaluate which part of healthcare items was changed. Changes in drug expenses and medical service expenses directly impacted by the policy are in line with expectations. The results indicate that the western drug expenses decreased significantly and service expenses increased significantly after the intervention. It was seen in most studies that after the elimination of drug markups, there was a significant reduction in drug expenses (2, 3, 15, 17, 30). In this reform, the prices of medical service items such as surgical operations and traditional Chinese medicine services that involved both higher skilled and more intensive labor input were raised, which more properly reflect the professional value and contribute to building a sustainable healthcare system (10).

This study provided additional corroborative evidence to demonstrate that the implementation of ZMDP was associated with a decrease in medicine expenses. However, healthcare providers still had an incentive to overprescribe drugs since they may receive kickbacks from pharmaceutical companies. A previous study demonstrated that larger quantities of drugs and more expensive drugs were prescribed (13). At the same time, there were significant spillover effects that increased other healthcare expenses, which undermined the impact of the policy. As shown in Table 3, confronted with a limit on revenue from Western drug sales, healthcare providers may face incentives to make up for revenue losses by increasing revenues from traditional Chinese medicine that was not covered by the reform. The unanticipated increase in Chinese medicine expenses failed to alleviate the patient financial burden effectively.

Consistent with previous studies (2, 31), the results showed that the number of inpatient admissions with LC increased after the ZMDP, which suggested the ZDMP promoted inpatient service utilization to an extent. Of note, some studies have shown that hospitals may increase revenue by increasing the number of medical consumables, imaging, and laboratory tests to offset the decline in drug expenditure (2, 11, 17, 18, 32). However, we found no indication that the ZMDP was associated with the increase in consumables, imaging, and laboratory test usage for inpatients diagnosed with IHD, CRF, or LC. In particular, the medical consumables usage of inpatients diagnosed with IHD and LC instantaneously decreased after implementing ZMDP. Unsurprisingly, the effects of the ZMDP varied in different scenarios. While the central government has issued broad guidelines for how the pricing reform should be designed and implemented, the local governments were given considerable discretion over the details of the design of the pricing reform. Local governments were responsible for conducting the progress evaluation for hospitals and monitoring the progress of the reform. Therefore, the effects of ZMDP were substantially associated with the government financial subsidies for hospitals and the appointment of hospital directors (15).

Although the increase in consumables, imaging, and laboratory test usage was not detectable, the unanticipated increase in Chinese medicine expenses failed to alleviate the patient financial burden effectively, which might be attributed to the partnership based on common interests formed by physicians and hospitals (4). In addition, the doctors have information superiority and hospitals monopolize the medical services market, the implementation of ZMDP alone may not be enough to reverse the profit-driven behavior of healthcare providers (15, 29). Healthcare provides confronting reductions in revenue under the policy might have increased their prescribing of other lucrative drugs, such as traditional Chinese medicine. The problems with the overprescribing drugs threaten to undermine the potential benefits by harming individuals and jeopardizing the financial stability of health systems (33, 34). The following reforms should be approached systematically. Given that healthcare providers' rewarding and incentive structures were directly affected by the payment policies (4), the most central task is to develop an optimal payment system or mechanism that aligns with the mutual interests of patients and physicians. The prior payment system employed fee-for-service (FFS), which created perverse incentives for ineffective medical practices. The payment methods such as Big Data Diagnosis-Intervention Packet (DIP) and Diagnosis-Related Groups (DRGs), are a necessary and feasible way to fully correct the perverse incentives for excessive treatment and overuse of expensive services (35, 36). Additionally, policymakers should pay attention to the synergy among different policies. Strengthening public hospital reform and strictly controlling performance indicators are warranted to regulate physicians' behavior. For example, clinical audits should give scope for further development in the means of patient quality of life, financial aspects of the hospital, etc.

Methodologically, interrupted time series analysis is the commonly used quasi-experimental design to evaluate the longitudinal effects of an intervention (37, 38). The paper shows how segmented regression analysis can be used to evaluate policy interventions intended to contain the rapid growth of medical expenditures and address the distorted cost structure. Another advantage of this study is that the study used more detailed individual-level data to analyze the various components of expenses whereby the change in hospitalization expenses can be analyzed more deeply. It also investigates the heterogeneous impact on three diseases in our sample. This study filled the research gap on the effect of ZMDP on Chinese medicine expenses. The unanticipated increase in Chinese medicine expenses and medical service expenses moderated the effect of containing healthcare expenses and relieving the financial burdens of patients. Additionally, this study using more detailed data directly observed the number of healthcare services received by patients and did not detect the supply-induced excessive healthcare service needs. Our findings are critical because they provide meaningful enlightenment for policy making.

There are several limitations to be aware of. First, the data of other possible explanatory variables such as the patient's disease severity, complications, and financial income were unavailable. We were unable to control a series of covariate effects to eliminate potential confounding effects. Second, due to the complex policy environment, it is difficult to measure the net effect of ZMDP. During the same period, several healthcare policies were implemented to promote the reform of public hospitals in Beijing. The interaction of these factors should be considered when exploring the impact of ZMDP. Third, our sample data were restricted to coverage from January 2016 to December 2017, so it is relatively short for using the ITS method and difficult to explore the long-term effect of ZMDP. Due to the unavailability of data, subgroups analysis stratified by the hospital levels was difficult to conduct, and more discussions about the effects of ZMDP on orienting the patients from higher-tiered hospitals to lower ones were not included in this study. Fourth, this study mainly focused on the impact of ZMDP on healthcare utilization and expenses. Of note, quality of service delivery has long been recognized as an equally important deterrent to service use (39). Reform should control the unreasonable growth of medical costs without compromising the quality of services or worsening health outcomes. Unfortunately, the data can't directly observe the quality of healthcare services received by patients, it can't test the impact on health outcomes. Future research is warranted to collect more data and conduct further analysis to address the important issues.

## Conclusion

The ZMDP was demonstrated to be effective in reducing drug expenses, and the effects on healthcare utilization varied across diseases type. Nevertheless, the increase in medical service and Chinese medicine expenses diminished the effect of containing healthcare expenses and relieving the financial burdens of patients. To improve patients' affordability, it is recommended that policymakers should take multiple and effective measures to reverse the profit-driven behavior. The experiences gained from the Beijing reform may help inform further reforms for other parts of China and other low- and middle-income countries with similar systems.

## Data availability statement

The original contributions presented in the study are included in the article/supplementary materials, further inquiries can be directed to the corresponding author.

## Author contributions

ZZ, JW, and LY were responsible for the conception and design of the study, acquisition, analysis of data, and drafted the initial draft. YS, JZ, PH, and LY reviewed and revised the manuscript. All authors reviewed and approved the final manuscript.

## Funding

This study was funded by the National Natural Science Foundation of China (Grant numbers 72174010, 71911530221, and 71673004).

## Conflict of interest

The authors declare that the research was conducted in the absence of any commercial or financial relationships that could be construed as a potential conflict of interest.

## Publisher's note

All claims expressed in this article are solely those of the authors and do not necessarily represent those of their affiliated organizations, or those of the publisher, the editors and the reviewers. Any product that may be evaluated in this article, or claim that may be made by its manufacturer, is not guaranteed or endorsed by the publisher.
